# Withering corolla remains on the calyx tube enhance reproductive success in *Oxalis stricta* L.

**DOI:** 10.1002/pei3.10014

**Published:** 2020-05-21

**Authors:** Akio Imamura, Kota Hariu

**Affiliations:** ^1^ Hokkaido University of Education Asahikawa City Japan

**Keywords:** accumulated effective temperature, corolla remain, petal‐removal experiment, reproductive success

## Abstract

Several flower traits can affect plant reproductive fitness via pollinator attraction, herbivory defense, and thermal regulation of the pistil. In this study, we focus on thermal regulation of the pistil after flowering. We experimentally investigated the functional significance of the withering corollas that remain attached to the calyx tubes of *Oxalis stricta* L. We studied thermal regulation of the pistil by removing corollas and comparing the plants with and without corollas, under regulated dark and light periods, with an ambient temperature during the dark period lower than that during the light period. In plants lacking corollas, the pistil temperature was significantly lower than in control plants (with intact corollas) by approximately 2°C. Although fruit set in the corolla‐removed plants was not significantly different from that in control plants, the temperature threshold for 50% fruition in the corolla‐removed plants was significantly higher than that in the controls. Furthermore, the seed number, total seed weight, and single‐grain weight were significantly lower in the corolla‐removed plants than in control plants. The estimated annual number of reproductive cycles (from June to October), total seed number, and total seed weights were also lower in corolla‐removed plants. These findings indicate that the withering corolla remains play a role in thermoregulation of the pistil, and thereby enhance reproductive success. Our study is the first to validate one of the assumed ecological roles of the withering remains of plant corollas.

## 1 INTRODUCTION

In plants, several traits can affect reproductive fitness after flowering, including pollinator attraction (Ida & Kudo, 2003; Kudo, 1995; Moyroud & Glover, 2016; Overland, 1960), herbivory defense (McCall & Irwin, 2006; Wu & Yahara, 2017), prevention of pollen loss (Bynum & Smith, 2001; von Hase, Cowling, & Ellis, 2006; Prokop, Jersáková, Fančovičová, & Pipíška, 2019), protection against pathogens (van Doorn & van Meeteren, 2003), and thermal regulation of the pistil (Abdusalam & Tan, 2014; Liu, Zhang, Ji, Zhang, & Wang, 2017; van der Kooi, Kevan, & Koski, 2019). Additionally, as indicated by van der Kooi et al. (2019), the question of whether flower closure is an adaptive trait that enhances reproductive success remains to be further explored.

The significance of plant movements was originally examined by Darwin (1880), who conducted detailed studies of leaf opening and closure and hypothesized that their ultimate function was nocturnal thermal maintenance facilitated by a reduction in the leaf surface area when the leaves were closed. As Darwin indicated, diurnal movements could also be related to plant thermal regulation. The ecological role of temporary flower closure with regards to thermal regulation and pollen viability has been previously studied. Prokop et al. (2019), for example, showed that flower closure increased pollen viability and stigma receptivity in *Crocus* species via thermal regulation, and Liu et al. (2017) have reported that petal closure is beneficial with respect to reproductive success in *Magnolia* species.

In this study, we focused on a further potential thermoregulatory mechanism associated flower reproduction, that is, the role played by closed and withering corolla remains after flowers have permanently closed. In some species, when the flowers close permanently, their petals begin to wither a few days after blooming. In this regard, Van Doorn (2001) categorized the cessation of flower life into two phases, senescence (withering) and abscission, which tend to show consistent patterns within families. Although flower closure and petal senescence/abscission after pollination, which shorten flower life, have been well studied (van Doorn & Kamdee, 2014), the research has focused primarily on proximate factors, such as the physiological aspects. Recently, however, Kyogoku, Kataoka, and Kondoh (2019) reported that the flowers of a diploid *Taraxacum* species rapidly close their heads in response to the landing of pollen, and also discuss the ecological aspects of this closure.

During the process of permanent flower closure, some species retain their withering corollas longer than others. However, the ecological function of the withered petals or corolla remains, and the reason why they remain attached to the calyx tube after pollination and during the fruiting period, have yet to be examined in detail. As flowers bear important reproductive organs, such as pistils and stamens, they are assumed to have evolved certain adaptive traits (Abdusalam & Tan, 2014), such as protection from airborne pathogens and frugivorous animals (van Doorn & van Meeteren, 2003; Wu & Yahara, 2017). Therefore, in this study, we sought to investigate whether the withering remains of corollas that are retained on the calyx tube have any ecological significance.

We hypothesized that the withering corolla remains have a universal function of maintaining constant pistil and ovule temperatures, which contributes to reducing the level of seed deterioration. We tested this hypothesis by performing experiments on *Oxalis stricta* L. (Oxalidaceae) to examine the effects on seed production of removing gamopetalous corollas from flowers.

## 2 MATERIALS AND METHODS

### 2.1 Plant material

**FIGURE 1:**
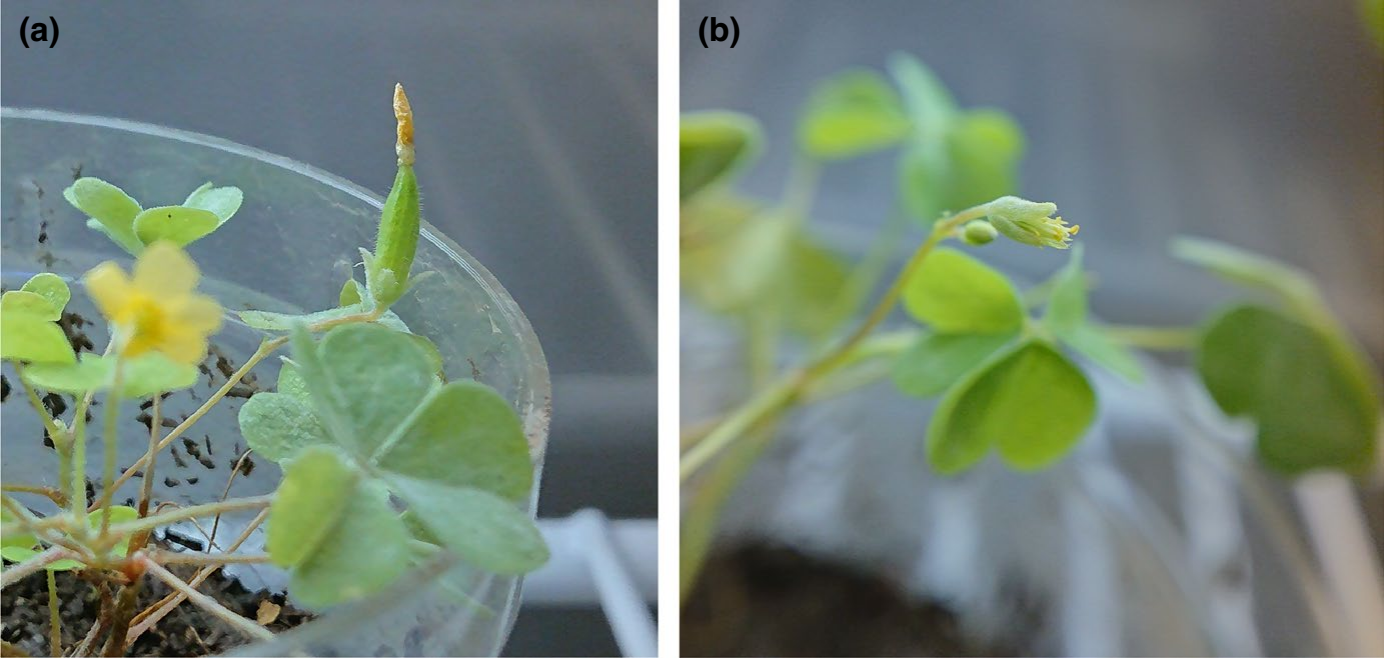
A flower of *Oxalis stricta.* (a) A flower with the withering corolla remains at the end of seed maturation. (b) A flower with petals removed. The individuals have long and short stamens and long pistils

We used *O. stricta*, a perennial herb that grows in circles in grasslands, as our experimental plant. It is widely distributed from temperate to subarctic zones in East Asia and eastern North America, and naturalized in Europe and Africa, flowering in June–October (Ohashi, Kadota, Murata, Yonekura, & Kihara, 2016b). *O. stricta* has a schizopetalous corolla of approximately 5 mm in diameter, which performs opening and closing movements. The flowers are hermaphroditic and have heteromorphous stamens, that is, five short and five long stamens. The long stamens, which are equal in length to the pistil, begin to self‐pollinate soon after the plant blooms. The plant produces one to three flowers per reproductive cycle. These cycles repeat sequentially per individual, with successive flowers blooming ca. 3–7 days after the preceding flowers have bloomed. However, the number of cycles per individual per year remains unknown. The corolla closes at night, under low temperatures, and in rainy weather conditions. These movements last for ca. 1–7 days, and the corolla falls off 3–16 days after blooming. The withering corolla remains attached during seed maturation (Figure 1a).

We selected eight patches from two sites in Asahikawa City, Hokkaido, Japan; seven patches in Hokkaido University of Education (43.787°N, 142.346°E) and one in Asahikawa City Northern Wild Plants Garden (43.790°N, 142.298°E). We collected eight *O. stricta* plants from each patch between July 29, 2018, and September 14, 2018, and for a total of 64 plants. They were planted in pots and subjected to acclimation that consisted of 4 hr of soaking in water in an incubator (MIR‐252; Sanyo Electric Co., Ltd.) at 25°C with a fluorescent light (FL15N; Panasonic Co., Ltd.). All flowering buds except one were picked from each plant before blooming, because if a plant has both damaged flowers (such as those with the corolla removed) and intact flowers, damaged flowers are naturally aborted in some plant species (Stephenson, 1981).

### 2.2 Laboratory experiments

Despite the plants favoring self‐pollination with the abovementioned longer stamens, we artificially self‐pollinated the plants with a small brush immediately after the flowers bloomed as the pollen of this species matures just after blooming to standardize the pollination conditions. The plant self‐pollinates soon after blooming, which is considered to be enhanced by the closing corolla. Thus, the corolla was removed using tweezers (Figure 1b) on the first day of blooming, just before the flower closed for the first time. The treated (corolla‐removed) and untreated or control (with intact corolla) plants from the same patch were cultivated together under the same day/night conditions. Sixty‐four plants were tested under two removal states and four temperature levels, and the experiment was carried out with eight replicates. The experiment lasted from the start of blooming to the end of the seed maturation phase, that is, 15–28 days. The flower buds, which emerged in just 3 days and were not the result of artificial self‐pollination, were picked immediately to avoid plants allocating resources toward them.

Throughout the experiment, the ambient temperature in the dark period was set to four different levels, that is, 23, 18, 13, and 8°C, using the abovementioned incubator. During the light period, the temperature was set to 25°C for all plants, and the plants were kept under a photoperiod of 12‐hr light and 12‐hr dark, starting at 19.00 and 07.00, respectively, to eliminate the effects of the circadian rhythm.

We measured the temperature of each pistil using an infrared thermometer (IR‐308; Custom Co., Ltd.) after every 4 hr for 24 consecutive hours (at 00.00, 04.00, 08.00, 12.00, 16.00, and 20.00, of which 08.00, 12.00, and 16.00 fall in the dark period). Pistil temperatures were measured, and the measurements were repeated twice in the middle of each flowering period. The closed corollas were temporarily opened with tweezers and their temperatures were measured using a thermometer.

We recorded seed setting in all the plants and calculated the total seed number and weight per flower, using a Mettler balance (METTLER AE260; Mettler Toledo Co., Ltd.). The single seed weight was calculated by dividing the total seed weight by the seed number for each plant. As the maximum number of flowers per blooming cycle per individual of *O. stricta* is three (Ohashi et al., 2016b), the estimated total seed number and weight in one blooming season (June–October) were calculated as 3 × total seed number per plant × number of reproductive cycles per year and 3 × seed weight per plant × number of reproductive cycles per year, respectively.

### 2.3 Statistical analyses

We used R 3.5.2 for Mac OSX (R Core Team, 2018) for all statistical analyses. Differences in ambient and pistil temperatures were tested using Welch's *t* test. Differences in fruit sets between the two corolla removal states were tested using the prop.test function in R, which tested the null hypothesis that the proportions (probabilities of success) in several groups were the same based on the Pearson's chi‐squared test, and the threshold temperature for 50% fruition was calculated using a logistic regression with a binomial distribution.

Differences in fruition rate, seed number and weight were analyzed using a generalized linear model (GLM) and likelihood ratio test with the research site (two sites), night temperature (four levels), and the corolla removal treatment (removed and intact) as the explanatory variables. The fruition rate, seed number, and seed weight was modeled using binomial distribution, Poisson distribution, and Gaussian distribution, respectively. Afterwards, differences in seed number and weight between corolla movement treatments were tested using pairwise Welch's *t* tests.

The hourly temperature data from June 1 to October 31 (153 days), which is the flowering season of *O. stricta*, were collected for all years from 1990 to 2018 from the Japan Meteorological Agency of Asahikawa (http://www.data.jma.go.jp/obd/stats/etrn/index.php?prec_no=12&block_no=47407&year=2018&month=06&day=01&view=) using the R package XML. We recorded the effective temperature (>5°C) during this period and calculated the accumulated degree hours (ADH; an alteration of the generally used accumulated degree days), which was designated as Td.

To estimate the number of reproductive cycles, we recorded the effective ambient temperature demand of each plant every hour between the blooming and seed maturity phase, and then pooled these data for all plants. This temperature was designated as Te (ADH for plants with the corolla removed) and Teʹ (ADH for plants with an intact corolla). During our study, it was observed that subsequent flowers bloomed approximately 3 or more days after the preceding flowers had bloomed. Hence, we assumed that the duration of our experimental period was equivalent to approximately half of the usual flowering and fruition cycle and calculated the number of reproductive cycles by dividing Td (ADH per year) by Te/2 or Te'/2. The differences between Te and Teʹ and in the number of reproductive cycles between treatments were tested using a pairwise Welch's *t* test.

## 3 RESULTS

Fruiting was observed in 24 of 64 *O. stricta* plants (nine corolla‐removed and 15 control plants). The mean periods for reaching seed maturity were 22.33 days (*SD* = 2.64) and 20.26 days (*SD* = 4.90) in corolla‐removed and control plants, respectively. The mean corolla‐fall‐off periods were 12.5 days after blooming (*SD* = 1.55) and 7.8 days before seed maturity (*SD* = 4.29) in control plants. One of the corolla‐removed plants and its control pair were excluded from the analyses as the corolla‐removed plant, at the end of its fruition, was damaged by a serious earthquake (i.e., 2018 Hokkaido Eastern Iburi Earthquake).

**FIGURE 2:**
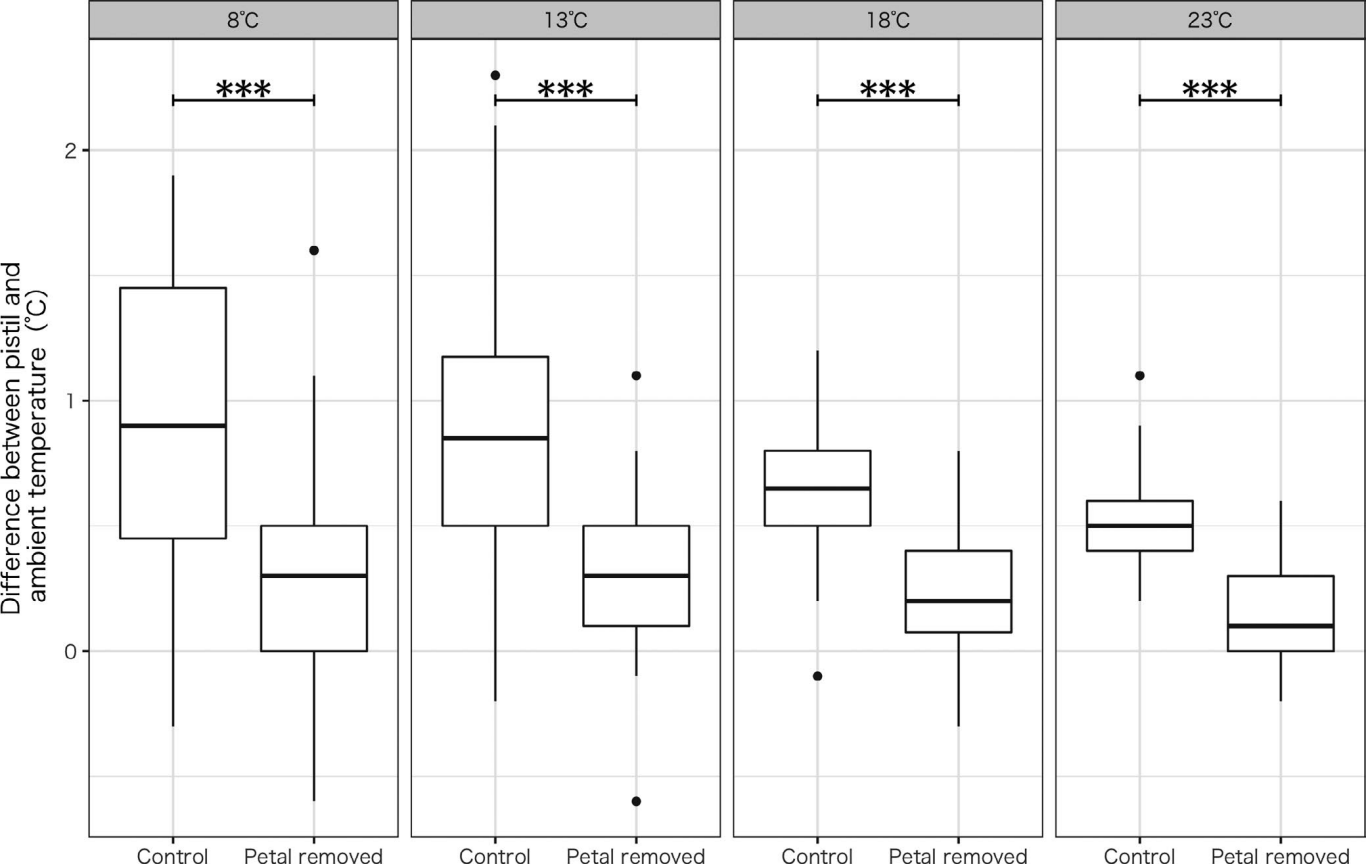
Difference between the pistil temperature of *Oxalis stricta* and ambient temperature for each nocturnal temperature level of the incubator. The difference between corolla‐removed and control individuals was significant by Welch's *t* test. ***p < .001

**FIGURE 3:**
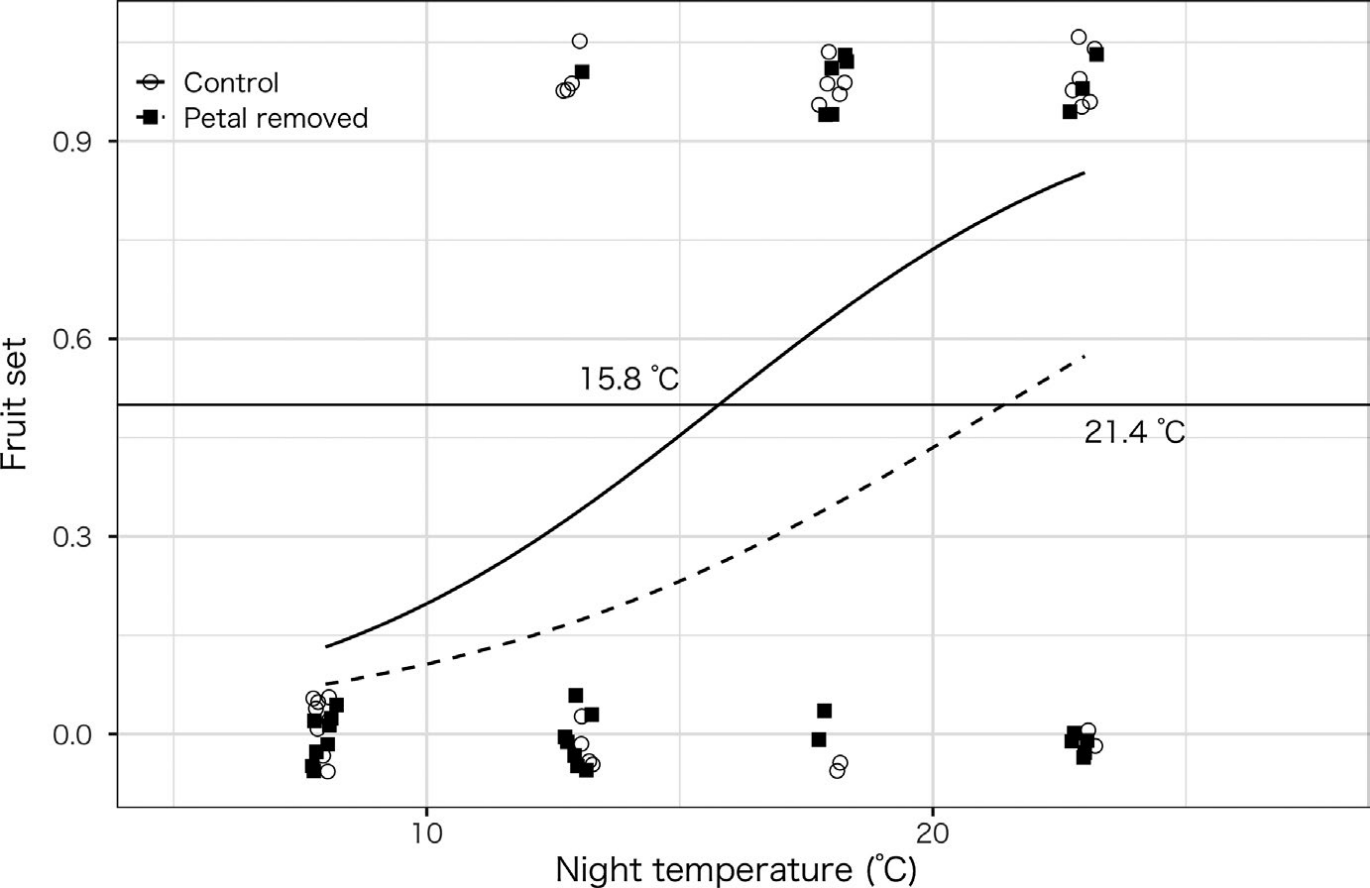
Threshold temperature of fruition of *Oxalis stricta* estimated for corolla‐removed and control individuals. The temperature at 50% fruition was estimated using a logistic regression model. Solid lines/open circles and dashed lines/closed squares indicate control and corolla‐removed individuals, respectively. Data points are jittered using R function

Pistil temperatures during the dark period were significantly lower in the corolla‐removed plants than those in the control plants (*t* test, *p* < .001; Figure 2). The fruition rate was not significantly different between corolla‐removed and control plants (prop.test, *p* = .12). However, the threshold temperature for 50% fruition in corolla‐removed plants was higher than that in control plants (21.4 and 16.3°C, respectively; Figure 3). Fruition rate was significantly and positively affected by the night temperature according to the likelihood ratio test (*p* < .001) although the rate was not significantly affected by the treatment (removed or intact, *p* = .06). Although no plants from the one patch in Asahikawa City Northern Wild Plants Garden fruited, the site effect was not statistically significant according to the test above (*p* = .99). Thus, the site effect was not included into the test below.

The seed number, total seed weight, and single‐grain weight, were significantly and negatively affected by the removal treatment (*p* < .001, *p* < .001, *p* < .05, respectively, by likelihood ratio test) and not by the night temperature (*p* = .178, *p* = .997, *p* = .128, respectively, by likelihood ratio test), according to GLMs with the removal treatment and the night temperature as explanatory variables.

We then tested the difference between the removal conditions by pairwise *t*‐test. The mean total seed number per plant was 8.44 (*n* = 9, *SD* = 3.36) and 21.2 (*n* = 15, *SD* = 9.54), and the mean total seed weight per plant was 0.0013 g (*SD* = 0.00054) and 0.0041 g (*SD* = 0.0018) in corolla‐removed and control plants, respectively. The seed number in control plants was significantly higher than that in corolla‐removed plants (*p* < .001; Table 1). The total and single seed weights in control plants were significantly higher than those in corolla‐removed plants as shown by pairwise Welch's *t* test (*p* < .001 and *p* < .05, respectively; Table 1).

**TABLE 1:** Results of petal_removal experiment for *Oxalis stricta*. Seed number and seed weight (g) were measured and weight per grain is calculated. The number of reproduction, total seed number, and total seed weight were estimated using meteorological data.

	Mean for control	*SD*	Mean for petals‐removed	*SD*	
Seed number per individual	21.2	9.54	8.444	3.358	***
Seed weight per individual	0.00408	0.00182	0.00132	0.000578	***
Calculated seed weight per grain	0.000197	0.00005	0.000158	0.0000333	*
Estimated number of reproduction per year	10.98	0.694	9.727	0.615	***
Estimated seed number per year per individual	698.319	44.178	246.412	15.589	***
Estimated seed weight per year per individual	0.1346	0.00852	0.0385	0.00244	***

* *p* < .05;

*** *p* < .001 by Whekh's *t* test.

**FIGURE 4:**
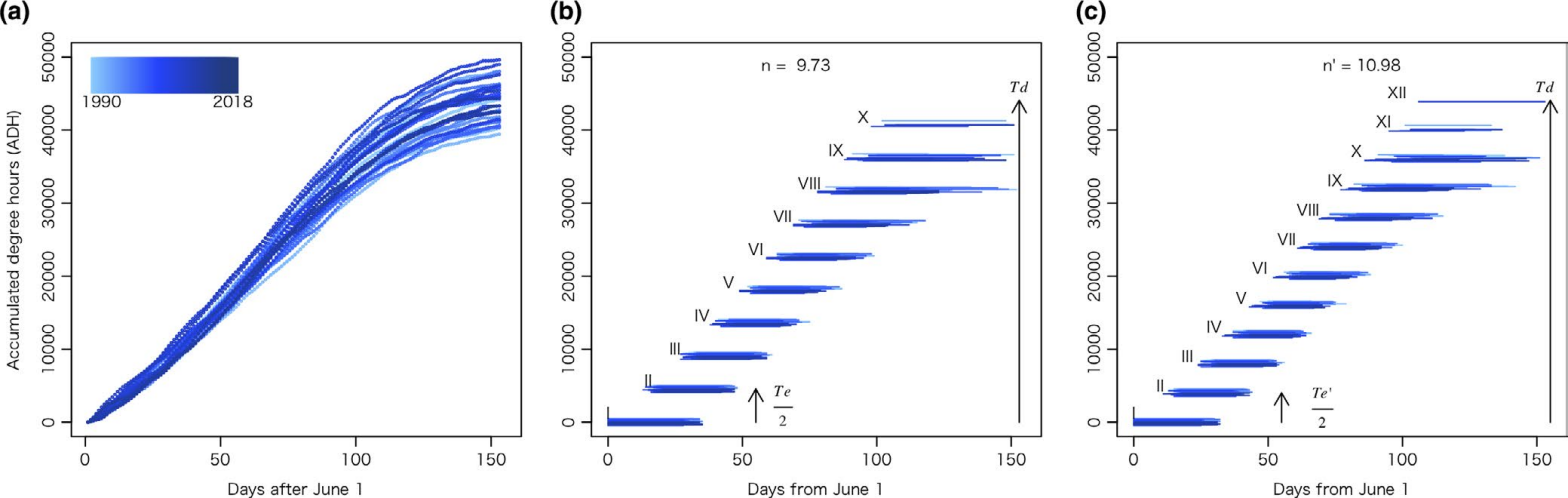
Estimated number of reproductive cycles in *Oxalis stricta* per year (June 1–Oct 30) using meteorological data from 1990 to 2018. The *y*‐axis denotes the accumulated degree hours (ADH). (a) ADH for each year using data from the Meteorological Agency of Asahikawa, Japan. (b) Estimated reproductive cycles for corolla‐removed plants (Td divided by Te/2; mean: *n* = 9.73). Te denotes the effective temperature demand for corolla‐removed individuals. The subsequent flower is assumed to bloom at the accumulation of half Te. (c) Estimated reproductive cycles for the control individuals (Td divided by Teʹ/2; mean: *nʹ* = 10.98). *Teʹ* denotes the effective temperature demand for the control plants. The subsequent flower is assumed to bloom when half Teʹ is accumulated. Td: ADH for each of the 29 years of meteorological data (mean for each year = 44,082 ADH)

Td in June − October was calculated from the meteorological data of 29 years (mean = 44,082 ADH, *SD* = 2,788; Figure 4a). The mean effective temperature demands, Te and Te', were 9,064.00 and 8,029.60 ADH, respectively, and Te was significantly higher than Te' as determined by pairwise Welch's *t* test (*p* < .001). The mean number of reproductive cycles calculated using Td was 9.72 (8.71–10.95; Figure 4b) and 10.98 (9.83–12.36; Figure 4c) in corolla‐removed and control plants, respectively, and the number of reproductive cycles was significantly higher in control plants than those in the corolla‐removed ones, as shown by pairwise Welch's *t* test (*p* < .001).

The estimated total seed number and weight during one blooming season in control plants were both significantly higher than those in corolla‐removed plants as determined by pairwise Welch's *t* test (*p* < .001, *p* < .001, Table 1).

## 4 DISCUSSION

As hypothesized, we found that pistil temperatures in corolla‐removed plants were significantly lower than those in control plants, indicating that withering corolla remains are effective in maintaining the pistil temperature. This suggests that the function of the retained corolla is to prevent pistil exposure to cold ambient air. This effect is more remarkable at lower ambient temperatures, when exposure could decrease pistil temperature. Therefore, this suggests corolla remains are beneficial and increase reproductive fitness. Regarding the cost of retaining the withered corolla, the cost of retaining dying or dead tissue is considered to be lower than that required for the maintenance of living tissue such as petals (Ashman & Schoen, 1997; Franchi, Nepi, & Pacini, 2014; He, Duan, Liu, & Smith, 2005).

As all other organs were intact in the corolla‐removed flowers and no additional flowers receiving reproductive resources were left on the plants, selective abortion was not activated in the corolla‐removed plants (Stephenson, 1981). Thus, fruition rate did not significantly differ between the corolla‐removed and control plants. However, the threshold temperature for 50% fruition in the corolla‐removed plants was higher than that in control plants.

The seed weight per grain and seed number per plant in corolla‐removed plants were lower than those in the control. This indicates the possible role of the withering corolla remains in the production of more and bulkier seeds by maintaining the pistil temperature, which is the most significant function of the withering corolla remains on flowers. Our results are consistent with preceding studies such as Wada (1998), Zhang, Ai, Yu, Wang, and Li (2010), and Liu et al. (2017), which reported that thermal keeping by heliotropism or petal closing enhance seed production.

Trade‐offs between the seed number and grain weight would suggest either more lighter seeds or fewer heavier seeds. Contrastingly, our results showed higher numbers of heavier seeds in the control plants. As the fruit set did not differ between experimental and control plants, the results were likely caused by differences in pollen‐tube growth or in maturation after fertilization.

We had expected the seed maturation in corolla‐removed plants to take longer than that in the control as the recorded Te was significantly higher than Te'. Thus, the potential number of reproductive cycles per year in corolla‐removed plants was estimated to be lower than that in the control. Consequently, the total number of seeds per year in corolla‐removed plants was also lower than that in the control plants.

Our results were derived from data of 64 assessed individuals of one plant species. However, the corolla removal treatment certainly affected plant reproductive fitness regarding the seed number, total seed weight, and single‐grain weight. Other plant taxa, such as *Trifolium* species of Fabaceae or *Mukia* species of Cucurbitaceae, are also known to retain their withering sepals or corolla during fruiting periods (Ohashi, Kadota, Murata, Yonekura, & Kihara, 2016a; Ohashi et al., 2016b), and their withered tepals could bear the same function suggested by the results of this study. Thus, our study was the first to validate one of the assumed ecological roles of withering corolla remains, that is, thermal maintenance of the pistil, which is used for increasing reproductive success. van Doorn (2001) has categorized the flower life cessation depending on senescence/abscission. However, the ecological difference between senescence and abscission, especially concerning the function of the corolla remains, has not yet been studied. Although the corolla remains might also have other functions, such as protecting the inner floral parts from the entry of airborne pathogens (van Doorn & van Meeteren, 2003) and frugivorous animals (e.g., slugs; Wu & Yahara, 2017), we conclude that the ecological function of thermal maintenance for seed maturation is thought to be universal in many plant lineages that include plants with withering corolla remains. Thus, our study on the corolla remains presents a new aspect of thermal ecology in flowers that was not previously mentioned in the literature.

## CONFLICT OF INTEREST

The authors have declared that no competing interest exists.

## AUTHOR CONTRIBUTIONS

A.I. conceived the study, conducted analyses, and wrote the manuscript. K.H. conducted experiments.

## Supporting information

Supplementary Material

Supplementary Material

## Data Availability

All relevant data are within the paper and its [Link], [Link] files.
